# Lipid-lowering therapy and LDL-C control for primary prevention in persons with diabetes across 90 health systems in the United States

**DOI:** 10.1016/j.ajpc.2023.100604

**Published:** 2023-11-12

**Authors:** Emily Decicco, Eric D. Peterson, Anand Gupta, Kristin Khalaf Gillard, Evelyn Sarnes, Ann Marie Navar

**Affiliations:** a University of Texas Southwestern Medical Center, Dallas, TX, USA; b Esperion Therapeutics, Inc., Ann Arbor, MI, USA

**Keywords:** Diabetes, Statin, Cholesterol, Primary prevention

## Abstract

**Objective:**

National guidelines recommend statin therapy for patients with type 2 diabetes. We assessed the extent of moderate- to high-intensity statin therapy utilization in community practice.

**Methods:**

We evaluated lipid-lowering therapy (LLT) and low-density lipoprotein cholesterol (LDL-C) levels at baseline and 1-year follow-up in patients aged 40–75 years with type 2 diabetes but without atherosclerotic cardiovascular disease (ASCVD), across 90 health systems in the United States participating in an electronic health record–derived dataset, Cerner Real-World Data. Multivariable logistic regression was used to evaluate factors associated with utilization of moderate- to high-intensity statin.

**Results:**

We identified 241,232 patients with type 2 diabetes (58.1 % on moderate- to high-intensity statin, 7.0 % on low-intensity statin, and 34.9 % on no statin). Predictors of moderate- to high-intensity statin therapy included retinopathy (adjusted odds ratio [aOR], 1.26; 95 % confidence interval [CI], 1.15–1.38), hypertension (aOR, 1.52; 95 % CI, 1.43–1.61), and stage 3 chronic kidney disease (aOR, 1.14; 95 % CI, 1.07–1.21). Women (aOR, 0.85; 95 % CI, 0.82–0.87), and those with rheumatoid arthritis (aOR, 0.79; 95 % CI, 0.71–0.87), psoriasis (aOR, 0.85; 95 % CI, 0.75–0.96), and hepatitis C (aOR, 0.40; 95 % CI, 0.39–0.46), had reduced odds of moderate- to high-intensity statin treatment. Utilization of ezetimibe was rare (2.0 %). LDL-C control was suboptimal at baseline (37.0 % and 27.9 % had LDL-C ≥100 mg/dL and <70 mg/dL, respectively). At 1-year follow-up, the rate of moderate- to high-intensity statin therapy utilization was 65.3 %.

**Conclusion:**

Increased efforts are needed to improve LDL-C control and LLT use for primary prevention of ASCVD in adults with type 2 diabetes, in particular among women and those with risk-enhancing inflammatory conditions.

## Highlights

• The study included 241,232 patients with diabetes (but without ASCVD).
• Among these patients, 58.1 % were on moderate- to high-intensity statin.
• Women were less likely to be on moderate- to high-intensity statin than men.
• LDL-C control was suboptimal, and use of non-statin lipid-lowering therapy was low.
• Efforts are needed to improve lipid management in patients with diabetes.

## Graphical abstract

**Figure:**
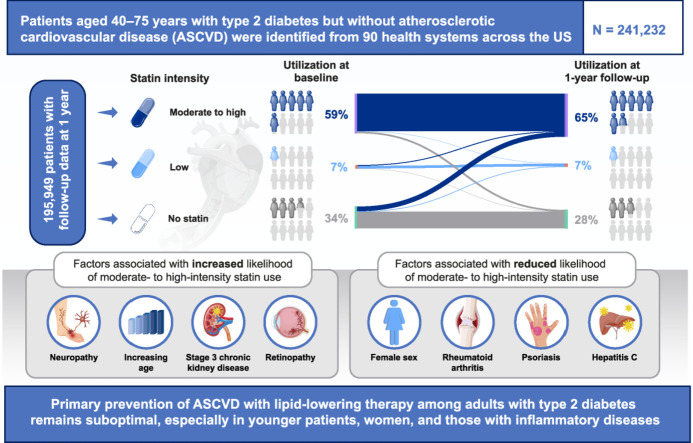
Central Illustration. Statin utilization and factors associated with likelihood of moderate- to high-intensity statin use in patients with type 2 diabetes. ASCVD, atherosclerotic cardiovascular disease.

Cardiovascular disease is the leading cause of death in patients with type 2 diabetes, which is an independent risk factor for atherosclerotic cardiovascular disease (ASCVD) [1], [2], [3], [4]. Among patients with type 2 diabetes, the long disease duration and complications such as retinopathy, neuropathy, and chronic kidney disease (CKD) may further increase the risk of cardiovascular disease [5], [6], [7]. The population-health implications for cardiovascular disease are substantial, with 11.3 % of the United States (US) population having diabetes and an additional 38 % having pre-diabetes [8]. Lipid lowering is a cornerstone of cardiovascular risk reduction, with statin therapy being recommended as first-line therapy for primary prevention of ASCVD in patients with type 2 diabetes [9,10]. For patients with diabetes aged 40–75 years, the 2019 American College of Cardiology (ACC)/American Heart Association (AHA) guidelines have a class I recommendation for a moderate- to high-intensity statin [9]. Similarly, the American Diabetes Association (ADA) issued a grade A recommendation for a moderate-intensity statin in people aged 40–75 years with type 2 diabetes [10]. The most recent 2019 ACC/AHA guidelines also recommend considering additional risk-enhancing factors for ASCVD that further support initiation of either a moderate- or high-intensity statin, including diabetes-specific risk factors and comorbid inflammatory diseases, such as rheumatoid arthritis, human immunodeficiency virus (HIV) infection, or hepatitis C [9]. Recommendations for non-statin lipid-lowering therapy (LLT) are limited in patients with diabetes. ADA guidelines recommend ezetimibe to achieve a goal of ≥50 % low-density lipoprotein cholesterol (LDL-C) reduction and advise against combination therapy with fibrates and niacin due to lack of clinical outcomes data [10]. The ACC/AHA guidelines do not include recommendations for non-statin therapy in persons with diabetes [9]. Despite these recommendations, multiple studies have shown that LLT remains underutilized in primary prevention [11], [12], [13], [14], [15]. Importantly, these studies are either population based, including patients who do not routinely seek medical care, or come from selected populations with limited generalizability. Understanding current patterns of LLT utilization among patients with diabetes seen in routine clinical practice offers an opportunity to target interventions to specific undertreated populations, and to highlight gaps in care. Therefore, we sought to evaluate LLT utilization for primary prevention of ASCVD, including changes in LLT over time, and factors associated with utilization of moderate- to high-intensity statin therapy in a large, multi-institutional cohort of adults with diabetes in the US.

## 1 Methods

This was a multicenter, retrospective, observational study utilizing Cerner Real-World Data (CRWD), a de-identified dataset derived from electronic health record (EHR) data from 90 participating health systems in the US. We identified patients aged 40–75 years with type 2 diabetes, without diagnostic codes for ASCVD, and who had an outpatient visit (index visit) between January 1, 2017, through December 31, 2018. Diabetes was defined based on a prior diagnosis of type 2 diabetes and prevalent use of at least 1 glucose-lowering medication. For each patient, diagnosis and procedure codes were evaluated for up to 3 years prior to the index visit to identify prior ASCVD or diabetes (see Supplemental Table S1 for code lists). Code types used included the International Classification of Diseases (ICD)-9, ICD-10, Current Procedural Terminology (CPT), Systematized Nomenclature of Medicine (SNOMED), and Healthcare Common Procedures Coding System (HCPCS) codes. Prior diagnoses were also used to identify prevalent hypertension, diabetes-specific risk factors (retinopathy and CKD), and inflammatory risk-enhancing conditions (rheumatoid arthritis, psoriasis, HIV, and hepatitis C).

Medication usage was determined from EHR medication lists, which included medication orders and self-reported medications by patients entered into their medical record. To ensure that we included patients with accurate medication data at baseline and follow-up, we restricted our sample to those with medication data for at least 1 year prior to the index visit and through at least 1-year follow-up. Using medication data, we determined statin and non-statin LLT use as of the index visit. Statin intensity was determined based on dose and statin type. We evaluated whether patients were on at least a moderate-intensity statin, based on guideline recommendations for moderate- or high-intensity statin in patients with diabetes [9]. Patients with a statin on their medication list without dose information were excluded from the analysis. To assess changes in medication over time, we identified patients in the cohort who had follow-up outpatient care in the system, defined as an outpatient visit at least 1 year after the index visit. Medication data at 1-year follow-up were evaluated to determine changes in therapy over time. Additional patient-level data included age, sex, race, ethnicity (recorded in the EHR, typically by self-report but may vary across health system), and obesity (body mass index [BMI] >30 kg/m^2^, using height and weight data from the most recent visit prior to baseline).

When laboratory data are available in the EHR as structured data, they are also included in CRWD. To evaluate LDL-C levels over time, we identified each patient's most recent LDL-C measurement using data for up to 24 months prior to the index visit as the “baseline” LDL-C level. If a patient had multiple prior LDL-C measurements, the LDL-C level closest to the index visit was considered as “baseline”. Next, we identified follow-up LDL-C levels up to 1 year following the index visit to determine the “follow-up” LDL-C level. For follow-up LDL-C, the last LDL-C measurement in the year following the index visit was used.

Patients receiving a moderate- to high-intensity dose of statin were compared with patients receiving no or low-intensity statin using the Wilcoxon rank sum test for continuous variables and Pearson's chi-squared test for categorical variables.

Univariable and multivariable logistic regression analyses were performed to determine the factors associated with utilization of moderate- to high-intensity statin therapy in patients with type 2 diabetes. Factors prespecified for inclusion in the model included age, sex, race, ethnicity, hypertension, rheumatoid arthritis, psoriasis, HIV, hepatitis C, retinopathy, neuropathy, and CKD stage based on estimated glomerular filtration rate. Odds ratios (ORs) were reported with 95 % confidence intervals (CIs) for both univariable and multivariable analyses. For age (a continuous variable), the linearity assumption was tested, and the relationship between age and statin utilization was non-linear (*p* <0.001), so age was modeled as a restricted cubic spline with 5 knots. To account for clustering at the health-system level, a Huber-White cluster sandwich estimator of variance was used. Because this study only included deidentified data, it was considered exempt from institutional review board (IRB) review by the University of Texas Southwestern IRB.

Data were licensed from Cerner Corporation for this study; therefore, are not publicly available.

## 2 Results

Between January 1, 2017 and December 31, 2018, 241,232 patients with diabetes and without diagnostic codes for ASCVD were identified across 90 US health systems. At baseline, 58.1 % (*n* = 140,234) of patients were on a moderate- to high-intensity statin, 7.0 % (*n* = 16,819) were on a low-intensity statin, and 34.9 % (*n* = 84,179) were on no statin. The median age was 60 years, 47.5 % were men, 78.2 % were White, and 84.3 % were non–Hispanic or Latino. Characteristics of the study population overall and stratified by statin intensity are shown in Table 1.

**Table 1:** Baseline Demographics and Clinical Characteristics in the Overall Study Population and Stratified by Baseline Statin Intensity.

		**Baseline statin intensity**		
**Characteristic**	**Overall (*N* = 241,232)**	**Moderate or high (*n* = 140,234)**	**Low (*n* = 16,819)**	**No statin (*n* = 84,179)**
**Age, median (IQR), years**	60 (53–67)	61 (54–67)	62 (54–68)	58 (51–65)
**Age group, *n* (%)**				
40–54 years	71,514 (29.6)	35,588 (25.4)	4281 (25.5)	31,645 (37.6)
55–64 years	88,531 (36.7)	52,922 (37.7)	6081 (36.2)	29,528 (35.1)
65–75 years	81,187 (33.7)	51,724 (36.9)	6457 (38.4)	23,006 (27.3)
**Sex, *n* (%)**				
Female	126,446 (52.5)	71,117 (50.8)	9483 (56.2)	45,846 (54.4)
Male	114,293 (47.5)	68,838 (49.2)	7293 (43.3)	38,162 (45.2)
Unknown	493	279	43	171
**Race, *n* (%)**				
American Indian or Alaska Native	1540 (0.7)	776 (0.6)	90 (0.6)	674 (0.9)
Black or African American	38,591 (17.2)	22,697 (17.4)	2447 (15.6)	13,447 (17.1)
Mixed	775 (0.3)	413 (0.3)	56 (0.4)	306 (0.4)
Other	7938 (3.5)	4520 (3.5)	530 (3.4)	2888 (3.7)
White	175,546 (78.2)	101,754 (78.2)	12,517 (80.0)	61,275 (78.0)
Unknown	16,842	10,074	1179	5589
**Ethnicity, *n* (%)**				
Hispanic or Latino	36,657 (15.7)	20,453 (15.0)	2937 (18.0)	13,267 (16.3)
Not Hispanic or Latino	197,059 (84.3)	115,496 (85.0)	13,399 (82.0)	68,164 (83.7)
Unknown	7516	4285	483	2748
**Laboratory tests/vital signs**				
Obesity (BMI ≥30 mg/kg^2^), *n* (%)	156,479 (64.9)	91,740 (65.4)	10,803 (64.2)	53,936 (64.1)
BMI, median (IQR), kg/m^2^	32 (27–38)	32 (27–38)	32 (27–38)	32 (27–38)
Systolic BP, median (IQR), mmHg	132 (121–145)	131 (120–144)	131 (121–144)	132 (122–146)
Diastolic BP, median (IQR), mmHg	79 (71–85)	78 (70–84)	78 (70–84)	80 (72–87)
Systolic BP >140 mmHg, *n* (%)	82,422 (34.2)	46,275 (33.0)	5461 (32.5)	30,686 (36.5)
Diastolic BP >90 mmHg, *n* (%)	36,644 (15.2)	18,929 (13.5)	2160 (12.8)	15,555 (18.5)
**HbA1c, *n* (%)**				
<5.7 % (<39 mmol/mol)	7229 (4.2)	3789 (3.7)	491 (4.0)	2949 (5.0)
5.7 % to <6.5 % (39 to <48 mmol/mol)	34,989 (20.3)	20,707 (20.4)	2785 (22.6)	11,497 (19.7)
6.5 % to <7.0 % (48 to <53 mmol/mol)	28,974 (16.8)	17,641 (17.4)	2177 (17.6)	9156 (15.7)
7.0 % to <8.0 % (53 to <64 mmol/mol)	41,395 (16.8)	25,229 (24.9)	3087 (25.0)	13,079 (22.4)
8.0 % to <10.0 % (64 to <86 mmol/mol)	36,911 (21.4)	21,765 (21.5)	2557 (20.7)	12,589 (21.5)
≥10.0 % (≥86 mmol/mol)	22,714 (13.2)	12,265 (12.1)	1253 (10.1)	9196 (15.7)
Unknown	69,020	38,838	4469	25,713
**ASCVD risk–enhancing inflammatory conditions, *n* (%)**				
Rheumatoid arthritis	3507 (1.5)	1864 (1.3)	262 (1.6)	1381 (1.6)
Psoriasis	3280 (1.4)	1796 (12.8)	231 (1.4)	1253 (1.5)
HIV	1075 (0.4)	572 (0.4)	58 (0.3)	445 (0.5)
Hepatitis C	3216 (1.3)	1276 (0.9)	183 (1.1)	1757 (2.1)
**Hypertension, *n (%)***	185,547 (76.9)	113,223 (80.7)	58,850 (80.1)	58,850 (69.9)
**Diabetes-specific ASCVD risk enhancers, *n* (%)**				
Retinopathy	8682 (3.6)	5615 (4.0)	657 (3.9)	2410 (2.9)
Neuropathy	28,297 (11.7)	17,228 (12.3)	2045 (12.2)	9024 (10.7)
**Creatinine, median (IQR), mg/dL**	0.87 (0.70–1.05)	0.89 (0.72–1.08)	0.87 (0.70–1.04)	0.83 (0.70–1.01)
LDL-C, median (IQR), mg/dL*	88 (67–113)	81 (62–105)	87 (69–108)	101 (81–123)
LDL-C categories, *n* (%)*				
<55 mg/dL	18,253 (12.8)	14,492 (16.6)	1015 (9.5)	2746 (6.1)
55–69 mg/dL	21,574 (15.1)	15,921 (18.2)	1686 (15.8)	3967 (8.8)
70–99 mg/dL	50,297 (35.2)	30,910 (35.4)	4391 (41.2)	14,996 (33.3)
100–129 mg/dL	31,873 (22.3)	15,175 (17.4)	2324 (21.8)	14,374 (31.9)
130–159 mg/dL	14,200 (9.9)	6963 (8.0)	877 (8.2)	6360 (14.1)
160–189 mg/dL	4809 (3.4)	2590 (3.0)	271 (2.5)	1948 (4.3)
≥190 mg/dL	1944 (1.4)	1205 (1.4)	86 (0.8)	653 (1.4)
Unknown	98,282	52,978	6169	39,135

⁎ LDL-C data are available for 142,950 patients and indicate the most recent LDL-C measurement up to 24 months prior to baseline. ASCVD, atherosclerotic cardiovascular disease; BMI, body mass index; BP, blood pressure; HbA1c, glycated hemoglobin; HIV, human immunodeficiency virus; IQR, interquartile range; LDL-C, low-density lipoprotein cholesterol.

In univariable analysis, increasing age and male sex were associated with increased odds of moderate- to high-intensity statin therapy (*p* <0.001). Differences in statin use were also seen by race and ethnicity, with lower odds of moderate- to high-intensity statin use seen in American Indian or Alaskan Native (OR, 0.71; 95 % CI, 0.63–0.81) and Hispanic or Latino patients (OR, 0.86; 95 % CI, 0.83–0.89). Black or African American patients were slightly more likely to receive moderate- to high-intensity statin than White patients (OR, 1.07; 95 % CI, 1.04–1.10; Supplemental Table S2).

**Fig. 1:**
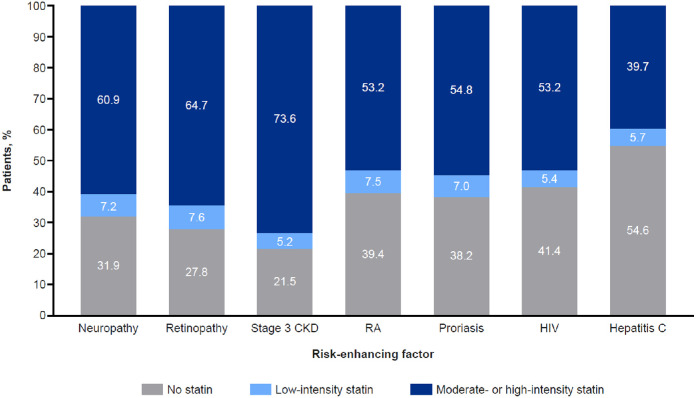
**Statin Utilization in Patient Subgroups at High Risk for ASCVD.** Proportion of patients on no, low-intensity, and moderate- or high-intensity statin by ASCVD risk factor. ASCVD, atherosclerotic cardiovascular disease; CKD, chronic kidney disease; HIV, human immunodeficiency virus; RA, rheumatoid arthritis.

Among high-risk patients, moderate- to high-intensity statin use was highest in those with diabetes-specific risk enhancers (neuropathy, retinopathy, and nephropathy) and lowest in those with inflammatory conditions (rheumatoid arthritis, psoriasis, HIV, and hepatitis C). Low-intensity statin use was uncommon (<7.5 %) in all groups (Fig. 1). In univariable analysis, diabetes-specific risk factors, including retinopathy, neuropathy, and nephropathy, were all associated with increased odds of moderate- to high-intensity statin use, while the inflammatory risk enhancers were associated with a decreased likelihood of moderate- to high-intensity statin use (*p* <0.001 for hepatitis C, rheumatoid arthritis, and psoriasis; *p* = 0.03 for HIV; Supplemental Table S2).

In multivariable modeling, older age was still associated with moderate- to high-intensity statin therapy. Above 65 years of age, increasing age was not associated with increased odds of moderate- to high-intensity statin therapy (adjusted OR [aOR], 0.98; 95 % CI, 0.91–1.04; Table 2 & Supplemental Figure S1). No association was seen between race or ethnicity and moderate- to high-intensity statin use (*p* >0.05; Table 2). Women were significantly less likely than men to be on a moderate- or high-intensity statin (aOR, 0.85; 95 % CI, 0.82–0.87). Statistically significant associations with decreased odds of moderate- to high-intensity statin therapy were maintained in multivariable analyses for rheumatoid arthritis (aOR, 0.79; 95 % CI, 0.71–0.87), psoriasis (aOR, 0.85; 95 % CI, 0.75–0.96), hepatitis C (aOR, 0.40; 95 % CI, 0.39–0.46), and hypertension (aOR, 1.52; 95 % CI, 1.43–1.61), but not HIV (*p* = 0.52), while retinopathy (aOR, 1.26; 95 % CI, 1.15–1.38) was associated with increased odds of moderate- to high-intensity statin therapy. All patients with CKD had increased odds of receiving moderate- to high-intensity statin therapy compared with those with normal renal function, but this was only statistically significant in those with stage 3 CKD (aOR, 1.14; 95 % CI, 1.07–2.19; Table 2).

**Table 2:** Factors Associated with Moderate- to High-Intensity Statin Use in Multivariable Modeling.

**Characteristic**	**aOR (95 % CI)**	***p* value**
**Age, years (aOR per 5-year increase)**		
<55	1.26 (1.17–1.33)	<0.001
55–60	1.12 (1.08–1.15)	
60–65	1.09 (1.05–1.14)	
≥65	0.98 (0.91–1.04)	
**Sex (Ref: male)**		
Female	0.85 (0.82–0.87)	<0.001
**Race (Ref: White)**		
American Indian or Alaska Native	0.82 (0.62–1.08)	0.15
Black or African American	1.08 (0.94–1.23)	0.27
Mixed	0.93 (0.83–1.05)	0.24
Other	1.08 (0.94–1.24)	0.29
**Ethnicity (Ref: not Hispanic or Latino)**		
Hispanic or Latino	0.94 (0.86–1.09)	0.39
**ASCVD risk enhancers (Ref: absence of factor)**		
Rheumatoid arthritis	0.79 (0.71–0.87)	<0.001
Psoriasis	0.85 (0.75–0.96)	0.01
HIV	0.94 (0.79–1.12)	0.52
Hepatitis C	0.40 (0.39–0.46)	<0.001
**Hypertension**	1.52 (1.43–1.61)	<0.001
**Diabetes-specific risk enhancers (Ref: absence of factor)**		
Retinopathy	1.26 (1.15–1.38)	<0.001
Neuropathy	1.06 (0.99–1.13)	0.06
**CKD (Ref: normal eGFR, >90** **mL/min/1.73 m^2^)**		
Stage 2 (eGFR, 60–89 mL/min/1.73 m^2^)	1.04 (0.98–1.10)	0.21
Stage 3 (eGFR, 30–59 mL/min/1.73 m^2^)	1.14 (1.07–1.21)	<0.001
Stage 4–5 (eGFR, <30 mL/min/1.73 m^2^)	1.08 (0.99–1.19)	0.08

aOR, adjusted odds ratio; ASCVD, atherosclerotic cardiovascular disease; CI, confidence interval; CKD, chronic kidney disease; eGFR, estimated glomerular filtration rate; HIV, human immunodeficiency virus; Ref, reference.

A total of 142,950 (59.3 %) patients in the overall cohort had LDL-C information prior to index visit. Among these patients, median LDL-C was 88 mg/dL; only 27.9 % had LDL-C <70 mg/dL and 37.0 % had LDL-C ≥100 mg/dL (Table 1). Patients on no statin had the highest LDL-C (median, 101 mg/dL; interquartile range [IQR], 81–123), followed by those on a low-intensity statin (median, 87 mg/dL; IQR, 69–108), while those on a moderate- or high-intensity statin had the lowest LDL-C (median, 81 mg/dL; IQR, 67–113). Among those on a moderate- to high-intensity statin, 34.9 % had LDL-C <70 mg/dL, compared with 14.9 % of those not on any statin (*p* <0.001) and 25.3 % of those on a low-intensity statin (*p* <0.001). Even among those on a moderate- to high-intensity statin, 29.8 % still had LDL-C  ≥100 mg/dL.

Compared with patients on moderate- to high-intensity statins, those on no statin had higher glycated hemoglobin (HbA1c) levels and higher rates of severely uncontrolled diabetes (15.7 % of those on no statin had HbA1c >10 % [>86 mmol/mol] vs. 12.1 % of those on moderate- or high-intensity statin, *p* ≤0.001). In contrast, among those on a low-intensity statin, 10.1 % had poorly controlled diabetes (HbA1c >10 % [>86 mmol/mol]) vs. 12.1 % of those on moderate- to high-intensity statin therapy (*p* <0.001). Similarly, blood pressure (BP) control was worse in those on no statin (36.5 % with systolic BP >140 mmHg and 18.5 % with diastolic BP >90 mmHg) compared with those on moderate- or high-intensity statin (33.0 % with systolic BP >140 mmHg and 13.5 % with diastolic BP >90 mmHg, *p* <0.001 for both).

Overall, utilization of non-statin LLT was low regardless of statin use at baseline (Supplemental Figure S2). Fibrates were the most commonly used non-statin LLT among those on no statin or on a low-intensity statin (6.3 % and 5.2 %, respectively). Ezetimibe use was low across all groups, including 2.2 % of those on moderate- to high-intensity statin, 1.8 % of those on no statin, and 1.4 % of those on low-intensity statin. Proprotein convertase subtilisin/kexin type 9 (PCSK9) inhibitors were used by ≤0.2 % of patients across all groups.

**Fig. 2:**
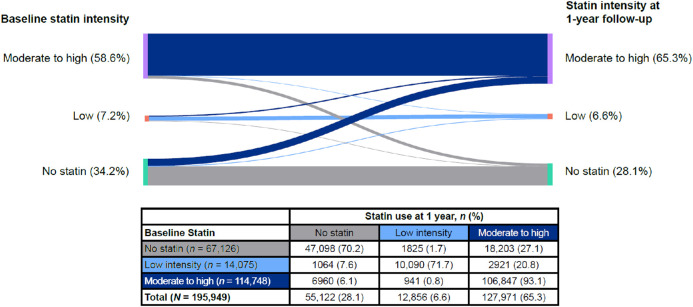
**Changes in Statin Utilization from Baseline to Year 1.** Statin utilization by dose intensity (no statin, low intensity, and moderate to high intensity) at baseline and at 1-year follow-up.

Of the 241,232 patients included in the study, 15,197 (6 %) had an ASCVD event within 1 year of the index visit and were excluded. An additional 554 patients with missing statin dose information and 29,532 patients without a follow-up outpatient visit were also excluded, resulting in a total of 195,949 patients included in the analysis of changes in statin use at 1-year follow-up. The vast majority of patients (*n* = 164,035 [83.7 %]) remained on the same statin intensity at 1-year follow-up as at baseline: 93.1 % of patients remained on a moderate- to high-intensity statin, 71.7 % of patients remained on a low-dose statin, and 70.2 % remained on no statin (Fig. 2). Only 26.0 % (21,124 of 81,201) of patients not on statin or on low-intensity statin at baseline had been initiated on a moderate- or high-intensity statin at follow-up. Among those on no statin and low-intensity statin, 27.1 % (*n* = 18,203) and 20.8 % (*n* = 2921), respectively, had initiated a moderate- to high-intensity statin at 1-year follow-up. However, a similar number of patients on a moderate- or high-intensity statin at baseline had either stopped statin therapy (*n* = 6960) or down-titrated to a low-intensity statin (*n* = 10,903) at follow-up. As a result, the overall use of moderate- or high-intensity statins was only slightly increased at 1 year, from 58.6 % (*n* = 114,748) to 65.3 % (*n* = 127,971).

Among the 142,950 patients with a “baseline” LDL-C measurement (within 2 years prior to the index visit), 89,120 (62.3 %) had an LDL-C measurement in the subsequent year. The median duration between the baseline and follow-up LDL-C measurements was 354 days (IQR, 255–434). In this population, 28.1 % of patients had LDL-C <70 mg/dL at baseline, which increased to 33.1 % at 1-year follow-up. Overall, the median absolute change in LDL-C in the population was −3 mg/dL (IQR, −18 to 11), representing <1 % relative change in LDL-C (Supplemental Table S3). At 1-year follow-up, LDL-C levels remained ≥100 mg/dL in 70.6 % of patients with LDL-C ≥100 mg/dL prior to the index visit. Among those with LDL-C <70 mg/dL prior to index visit, LDL-C increased to 70–99 mg/dL in 18.9 % of patients and to ≥100 mg/dL in 5.4 % of patients at follow-up (Supplemental Table S4).

## 3 Discussion

Despite the proven efficacy and safety of statins, among nearly 250,000 persons with diabetes from 90 health systems across the US, over 40 % were not on at least moderate-intensity statin therapy, and one-third were on no statin at all. Younger patients, women, and those with inflammatory conditions posing increased risk for ASCVD were among those least likely to receive moderate- to high-intensity statin therapy, and no differences were observed in utilization of moderate- to high-intensity statin therapy according to race or ethnicity. Among those receiving follow-up care in the same health system, statin therapy remained largely unchanged. LDL-C control was also suboptimal, as over one-third had LDL-C ≥100 mg/dL at baseline. Despite the observed high LDL-C levels, utilization of non-statin LLT was low. Collectively, these findings suggest that there remain large gaps in the clinical application of guideline-based LLT for primary prevention in persons with diabetes.

Our findings from a very large contemporary cohort are generally consistent with those from previous studies that included older, smaller, or more selected patient cohorts [13], [14], [15], demonstrating that use of LLT for primary prevention among persons with type 2 diabetes has improved little over time. In addition, our study population included patients who had been followed for at least a year in the same health system, the vast majority of whom had follow-up 1 year later. Because our population included only those with access to healthcare, rates of statin utilization were higher than those found in national cross-sectional survey data. In the National Health and Nutrition Examination (NHANES) survey from 2011 to 2015, only 49.5 % of persons with diabetes aged 40–75 years reported taking a statin [14], which is slightly lower than the 65.1 % found in our study. In the Patient and Provider Assessment of Lipid Management (PALM) registry, 70.1 % of persons with diabetes were treated with a statin, which is slightly higher than what we found, likely due to a patient consent requirement in PALM, leading to selection bias [16].

Our study found significant disparities in statin utilization according to age and sex. Women were less likely than men to be treated with moderate- to high-intensity statin therapy, which is consistent with previous reports showing sex-based differences in guideline-based LLT utilization [17]. In PALM, women were less likely to have been offered a statin and more likely to have declined or discontinued a statin than men [18]. We also found decreased statin utilization in younger patients. This represents a substantial missed opportunity because initiation of LLT earlier in life results in longer duration of therapy and greater lifetime absolute benefit [19].

Unlike some other studies that found lower rates of statin utilization in Black and Hispanic persons [15,[20], [21], [22]], we found no differences in statin utilization by race or ethnicity. There are several possible reasons for this discrepancy. First, our study selected for patients with routine access to healthcare, eliminating any disparities due to healthcare access. Second, our study used race and ethnicity data from the EHR, inaccuracies in which could bias any finding towards the null [23].

Patients with chronic inflammatory conditions such as HIV, hepatitis C, rheumatoid arthritis, and psoriasis have higher ASCVD risk compared with the general population, and statins can help lower risk in those with inflammatory disease [24], [25], [26], [27], [28], [29], [30], [31]. Despite being at increased risk, patients with chronic inflammatory conditions were significantly less likely to receive moderate- to high-intensity statin therapy than those without. Though statins are safe in patients with compensated chronic liver disease, including hepatitis C, concerns regarding hepatotoxicity may limit utilization of statins in this population [32,33]. Reasons for underutilization in patients with rheumatoid arthritis and psoriasis may relate to concerns regarding statin-associated muscle symptoms and/or lack of recognition of these conditions as ASCVD risk enhancers. Efforts to improve statin utilization in patients with inflammatory conditions should emphasize the increase in ASCVD risk associated with these conditions and the safety of statins in these populations.

In contrast to the relative undertreatment of chronic inflammatory diseases, patients with diabetes-specific risk enhancers indicating more severe or longstanding diabetes, such as retinopathy and CKD, were more likely to receive statin therapy consistent with guideline recommendations in multivariable modeling. This suggests that providers may be more willing to prescribe statin therapy to patients with more severe diabetes, and/or those with more severe disease are more willing to take therapy. Nevertheless, among patients with retinopathy, 27.8 % were not on any statin and 7.6 % were only on a low-intensity statin. Some patients may choose to not take a statin after a clinician–patient conversation, and others may not be eligible due to limited life span, statin intolerance, or drug–drug interactions; therefore, 100 % statin use is not achievable. However, given the high rate of cardiovascular events in persons with diabetes and complications [34], significant room for improvement appears to exist for statin utilization to prevent cardiovascular events.

We also found significant clinical inertia, with relatively low rates of statin up-titration over time. At 1 year, statin initiation or up-titration was uncommon: even among those within the same health system, only 27.1 % of those not on a statin and 20.8 % of those on a low-intensity statin were on a high-intensity statin at 1-year follow-up. Reassuringly, 93.1 % of those on a moderate- to high-intensity statin at baseline remained on a moderate- to high-intensity dose at 1-year follow-up. Thus, even small improvements in the rate of statin initiation and intensification in patients with diabetes are likely to lead to sustained improvements in overall lipid management at the population level over time.

Not all patients tolerate statins, and some patients may decline them due to fears about side effects [35]. Although guidelines do not generally recommend low-intensity statins for primary prevention in patients with diabetes, low-intensity statins are an option for those unable to tolerate higher doses. Additionally, non-statin LLT should be considered as an adjunct therapy. In our study, non-statin LLT use was low, and less than 10 % of patients were taking a low-intensity statin. However, those on a low-intensity statin had higher rates of BP control and lower rates of elevated HbA1c than those on high-intensity statin. This may indicate that those on a low-intensity statin are actually more engaged with their health and risk-factor management than the general population with diabetes. However, despite having better glycemic and BP control, those on a low-intensity statin had higher LDL-C levels than those on high-intensity statin. For those on a low-intensity statin willing to have therapy intensified, statin up-titration or addition of a non-statin LLT should be considered to maximize LDL-C lowering and reduce cardiovascular risk. Importantly, patients on moderate-intensity statins may still be undertreated, especially if they are considered high risk.

Evidence of efficacy for non-statin LLT is limited in primary prevention populations at high cardiovascular risk, including patients with type 2 diabetes. Cardiovascular risk reduction with ezetimibe and PCSK9 inhibitors has only been evaluated in patients with established ASCVD [36,37]. Ezetimibe has been shown to lower cardiovascular risk in patients with diabetes and established ASCVD when added to a statin [37] and is available at low cost, even to those without insurance. In our population, even among patients not on a statin, only 2 % were taking ezetimibe. In contrast, although several studies have shown that fibrates do not lower cardiovascular risk in patients with diabetes [38], [39], [40], fibrates were the leading non-statin LLT utilized in our population, used by 6.3 % of those not on a statin. Fortunately, new therapeutic options are available for primary prevention in patients with diabetes. Bempedoic acid was recently shown to lower the risk of major cardiovascular events in statin-intolerant patients, including high-risk primary prevention patients with type 2 diabetes [41].

Our study has several limitations inherent to its retrospective, observational design using EHR data. The generalizability of our findings depends on the representativeness of the participating health systems in the dataset. For example, only 0.7 % of patients in our dataset identified as American Indian or Alaska Native, compared with 1.3 % of the overall US population (2022) [42]. Furthermore, the proportion of patients in our dataset who identified as Black or African American (17.2 %) is higher than in the overall US population (13.6 %) [42]. CRWD does not include those institutionalized, those who receive care exclusively at the Veterans Affairs, Department of Defense, and Indian Health Services, and likely underrepresents these populations’ contributions. Medication information included self-reported and prescribed medications but did not include fill data; thus, our results may overestimate true statin utilization. However, our approach allows for capture of statin prescriptions that patients may pay for out of pocket, unlike claims data, which only capture prescriptions filled with insurance. Furthermore, lipid test results from external laboratories or other health systems were unavailable in our dataset. We also did not have access to pretreatment LDL-C levels, so we were unable to determine the degree to which LDL-C levels influenced initial treatment decisions. Finally, patients were required to have 2 years of data within the same health system, likely leading to a selection bias for those with higher healthcare access and utilization.

Statin therapy for primary prevention of ASCVD in patients with diabetes is underutilized, particularly among younger patients, women, and those with ASCVD risk–enhancing inflammatory diseases (**Central Illustration**). Most patients stayed on the same intensity statin at 1-year follow-up, and non-statin LLT use was very low overall. Over 1 in 3 patients with diabetes had LDL-C ≥100 mg/dL, suggesting the need for more aggressive interventions to target therapeutic inertia around lipid management and improve cardiovascular risk reduction in patients with diabetes.

## 4 Declaration of generative AI and AI-assisted technologies in the writing process

The authors declare that no AI-assisted technology was used during the writing process.

## Funding

This study was supported by Esperion Therapeutics, Inc.

## Author contributions

**Emily Decicco:** Conceptualization, Methodology, Writing - original draft, Writing - review & editing. **Eric D. Peterson:** Conceptualization, Funding acquisition, Methodology, Supervision, Writing - review & editing. **Anand Gupta:** Data curation, Formal analysis, Methodology, Project administration, Writing - review & editing. **Kristin Khalaf Gillard:** Conceptualization, Methodology, Resources, Writing - review & editing. **Evelyn Sarnes:** Conceptualization, Methodology, Resources, Writing - review & editing. **Ann Marie Navar:** Conceptualization, Data curation, Funding acquisition, Methodology, Project administration, Supervision, Writing - original draft, Writing - review & editing.

The authors take full responsibility for the content of this work. Study sponsor–employed authors contributed to the study conceptualization and manuscript writing. However, the ultimate interpretation of the data, manuscript presentation, and decision to publish were retained by the study senior author, Dr. Ann Marie Navar.

## Declaration of Competing Interest

The authors declare the following financial interests/personal relationships which may be considered as potential competing interests: Eric D. Peterson reports a relationship with Amgen that includes: consulting or advisory and funding grants. Eric D. Peterson reports a relationship with Esperion Therapeutics, Inc. that includes: consulting or advisory and funding grants. Eric D. Peterson reports a relationship with Janssen that includes: consulting or advisory and funding grants. Eric D. Peterson reports a relationship with Novo Nordisk that includes: consulting or advisory. Eric D. Peterson reports a relationship with Bristol Myers Squibb that includes: funding grants. Kristin Khalaf Gillard reports a relationship with Esperion Therapeutics, Inc. that includes: employment and equity or stocks. Evelyn Sarnes reports a relationship with Esperion Therapeutics, Inc. that includes: employment and equity or stocks. Ann Marie Navar reports a relationship with AstraZeneca that includes: consulting or advisory. Ann Marie Navar reports a relationship with Bayer that includes: consulting or advisory. Ann Marie Navar reports a relationship with Boehringer Ingelheim that includes: consulting or advisory. Ann Marie Navar reports a relationship with Esperion Therapeutics, Inc. that includes: consulting or advisory and funding grants. Ann Marie Navar reports a relationship with Janssen that includes: consulting or advisory and funding grants. Ann Marie Navar reports a relationship with Eli Lilly that includes: consulting or advisory. Ann Marie Navar reports a relationship with Merck that includes: consulting or advisory. Ann Marie Navar reports a relationship with NewAmsterdam Pharma that includes: consulting or advisory. Ann Marie Navar reports a relationship with Novartis that includes: consulting or advisory. Ann Marie Navar reports a relationship with Novo Nordisk that includes: consulting or advisory. Ann Marie Navar reports a relationship with Pfizer that includes: consulting or advisory. Ann Marie Navar reports a relationship with Silence Therapeutics that includes: consulting or advisory. Ann Marie Navar reports a relationship with Amgen that includes: funding grants. Ann Marie Navar reports a relationship with Bristol Myers Squibb that includes: funding grants. Emily Decicco and Anand Gupta have no relevant competing interests to declare.

## Appendix Supplementary materials

Image, application 1
