# Supplementary material for: Lipid-lowering therapy and LDL-C control for primary prevention in persons with diabetes across 90 health systems in the United States
Source: Am J Prev Cardiol. 2023 Nov 12;16:100604. doi: 10.1016/j.ajpc.2023.100604 (PMC10757181; doi:10.1016/j.ajpc.2023.100604)
Supplement: Supplementary file 1 [file mmc1.docx]

**SUPPLEMENT**

**TABLES**

**Table S1. Codes Utilized to Identify Clinical Conditions Based on Electronic Health Record Data**

| **Disease** | **Code type** | **Codes** |
| --- | --- | --- |
| **ASCVD** |  |  |
| Acute myocardial infarction | ICD-9-CM | "410", "410.0", "410.00", "410.01", "410.02", "410.1", "410.10", "410.11", "410.12", "410.2", "410.20", "410.21", "410.22", "410.3", "410.30", "410.31", "410.32", "410.4", "410.40", "410.41", "410.42", "410.5", "410.50", "410.51", "410.52", "410.60", "410.6", "410.61", "410.62", "410.70", "410.7", "410.71", "410.72", "410.8", "410.80", "410.81", "410.82", "410.90", "410.9", "410.91", "410.92" |
|  | ICD-10-CM | "I21", "I21.0", "I21.01", "I21.02", "I21.09", "I21.1", "I21.11", "I21.19", "I21.2", "I21.21", "I21.29", "I21.3", "I21.4", "I21.9", "I21.A", "I21.A1", "I21.A9", "I22", "I22.0", "I22.1", "I22.2", "I22.8", "I22.9" |
| Old myocardial infarction | ICD-9-CM | "411.0", "412", "429.79" |
|  | ICD-10-CM | "I23.0", "I23.1", "I23.2", "I23.3", "I23.4", "I23.5", "I23.6", "I23.8", "I24.1", "I25.2", "I23.7", "I23" |
| Prior CABG | CPT (HCPCS Level I) | "33508", "33510", "33511", "33512", "33513", "33514", "33516", "33517", "33518", "33519", "33521", "33522", "33523", "33530", "33533", "33534", "33535", "33536", "33572", "35500", "35572", "35600" |
|  | HCPCS Level II | "S2205", "S2206", "S2207", "S2208", "S2209" |
|  | ICD-10-PCS | "0210083", "0210088", "0210089", "021008C", "021008F", "021008W", "0210093", "0210098", "0210099", "021009C", "021009F", "021009W", "02100A3", "02100A8", "02100A9", "02100AC", "02100AF", "02100AW", "02100J3", "02100J8", "02100J9", "02100JC", "02100JF", "02100JW", "02100K3", "02100K8", "02100K9", "02100KC", "02100KF", "02100KW", "02100Z3", "02100Z8", "02100Z9", "02100ZC", "02100ZF", "0210344", "02103D4", "0210444", "0210483", "0210488", "0210489", "021048C", "021048F", "021048W", "0210493", "0210498", "0210499", "021049C", "021049F", "021049W", "02104A3", "02104A8", "02104A9", "02104AC", "02104AF", "02104AW", "02104D4", "02104J3", "02104J8", "02104J9", "02104JC", "02104JF", "02104JW", "02104K3", "02104K8", "02104K9", "02104KC", "02104KF", "02104KW", "02104Z3", "02104Z8", "02104Z9", "02104ZC", "02104ZF", "0211083", "0211088", "0211089", "021108C", "021108F", "021108W", "0211093", "0211098", "0211099", "021109C", "021109F", "021109W", "02110A3", "02110A8", "02110A9", "02110AC", "02110AF", "02110AW", "02110J3", "02110J8", "02110J9", "02110JC", "02110JF", "02110JW", "02110K3", "02110K8", "02110K9", "02110KC", "02110KF", "02110KW", "02110Z3", "02110Z8", "02110Z9", "02110ZC", "02110ZF", "0211344", "02113D4", "0211444", "0211483", "0211488", "0211489", "021148C", "021148F", "021148W", "0211493", "0211498", "0211499", "021149C", "021149F", "021149W", "02114A3", "02114A8", "02114A9", "02114AC", "02114AF", "02114AW", "02114D4", "02114J3", "02114J8", "02114J9", "02114JC", "02114JF", "02114JW", "02114K3", "02114K8", "02114K9", "02114KC", "02114KF", "02114KW", "02114Z3", "02114Z8", "02114Z9", "02114ZC", "02114ZF", "0212083", "0212088", "0212089", "021208C", "021208F", "021208W", "0212093", "0212098", "0212099", "021209C", "021209F", "021209W", "02120A3", "02120A8", "02120A9", "02120AC", "02120AF", "02120AW", "02120J3", "02120J8", "02120J9", "02120JC", "02120JF", "02120JW", "02120K3", "02120K8", "02120K9", "02120KC", "02120KF", "02120KW", "02120Z3", "02120Z8", "02120Z9", "02120ZC", "02120ZF", "0212344", "02123D4", "0212444", "0212483", "0212488", "0212489", "021248C", "021248F", "021248W", "0212493", "0212498", "0212499", "021249C", "021249F", "021249W", "02124A3", "02124A8", "02124A9", "02124AC", "02124AF", "02124AW", "02124D4", "02124J3", "02124J8", "02124J9", "02124JC", "02124JF", "02124JW", "02124K3", "02124K8", "02124K9", "02124KC", "02124KF", "02124KW", "02124Z3", "02124Z8", "02124Z9", "02124ZC", "02124ZF", "0213083", "0213088", "0213089", "021308C", "021308F", "021308W", "0213093", "0213098", "0213099", "021309C", "021309F", "021309W", "02130A3", "02130A8", "02130A9", "02130AC", "02130AF", "02130AW", "02130J3", "02130J8", "02130J9", "02130JC", "02130JF", "02130JW", "02130K3", "02130K8", "02130K9", "02130KC", "02130KF", "02130KW", "02130Z3", "02130Z8", "02130Z9", "02130ZC", "02130ZF", "0213344", "02133D4", "0213444", "0213483", "0213488", "0213489", "021348C", "021348F", "021348W", "0213493", "0213498", "0213499", "021349C", "021349F", "021349W", "02134A3", "02134A8", "02134A9", "02134AC", "02134AF", "02134AW", "02134D4", "02134J3", "02134J8", "02134J9", "02134JC", "02134JF", "02134JW", "02134K3", "02134K8", "02134K9", "02134KC", "02134KF", "02134KW", "02134Z3", "02134Z8", "02134Z9", "02134ZC", "02134ZF" |
|  | ICD-9-CM | "36.03", "36.1", "36.10", "36.11", "36.12", "36.13", "36.14", "36.15", "36.16", "36.17", "36.19", "36.2" |
|  | SNOMED CT | "10190003", "10326007", "119563008", "119565001", "14201006", "14323007", "149169006", "149173009", "149174003", "149183008", "17073005", "175006004", "175014005", "175021005", "175046005", "232717009", "232719007", "232720001", "232721002", "232722009", "232723004", "232724005", "265481001", "287277008", "29819009", "34147004", "359597003", "359601003", "39202005", "39724006", "405598005", "405599002", "589871000000109", "63077009", "67166004", "736967001", "736970002", "736971003", "736972005", "736973000", "74371005", "82247006", "90205004", "90487008" |
| Prior CABG diagnosis | ICD-10-CM | "Z95.1", "Z95.5", "I25.700", "I25.701", "I25.708", "I25.709", "I25.710", "I25.711", "I25.718", "I25.719", "I25.720", "I25.721", "I25.728", "I25.729", "I25.730", "I25.731", "I25.738", "I25.739", "I25.790", "I25.791", "I25.798", "I25.799", "I25.810", "I25.7", "I25.70", "I25.71", "I25.72", "I25.76", "I25.79", "I25.73" |
|  | ICD-9-CM | "V45.81", "414.02", "414.03", "414.04", "414.05" |
|  | SNOMED CT | "130541000119100", "139011000119104", "307765003", "316095000", "316096004", "316107000", "316868006", "316869003", "399261000", "400941000000105", "453291000000107", "469031000000107", "469611000000108", "15960381000119109", "15960541000119107", "15960581000119102", "15960661000119107", "15960781000119108", "371810009", "737276005", "251024009", "251025005" |
| Prior PCI procedure | CPT (HCPCS Level I) | "1021163", "92920", "92921", "92924", "92925", "92928", "92929", "92933", "92934", "92937", "92938", "92941", "92943", "92944", "92973", "92975", "92977", "92980", "92981", "92982", "92984", "92995", "92996" |
|  | HCPCS Level II | "C9600", "C9601", "C9602", "C9603", "C9604", "C9605", "C9606", "C9607", "C9608", "G0290", "G0291" |
|  | ICD-10-PCS | "0270046", "027004Z", "0270056", "027005Z", "0270066", "027006Z", "0270076", "027007Z", "02700D6", "02700DZ", "02700E6", "02700EZ", "02700F6", "02700FZ", "02700G6", "02700GZ", "02700T6", "02700TZ", "02700Z6", "02700ZZ", "0270346", "027034Z", "0270356", "027035Z", "0270366", "027036Z", "0270376", "027037Z", "02703D6", "02703DZ", "02703E6", "02703EZ", "02703F6", "02703FZ", "02703G6", "02703GZ", "02703T6", "02703TZ", "02703Z6", "02703ZZ", "0270446", "027044Z", "0270456", "027045Z", "0270466", "027046Z", "0270476", "027047Z", "02704D6", "02704DZ", "02704E6", "02704EZ", "02704F6", "02704FZ", "02704G6", "02704GZ", "02704T6", "02704TZ", "02704Z6", "02704ZZ", "0271046", "027104Z", "0271056", "027105Z", "0271066", "027106Z", "0271076", "027107Z", "02710D6", "02710DZ", "02710E6", "02710EZ", "02710F6", "02710FZ", "02710G6", "02710GZ", "02710T6", "02710TZ", "02710Z6", "02710ZZ", "0271346", "027134Z", "0271356", "027135Z", "0271366", "027136Z", "0271376", "027137Z", "02713D6", "02713DZ", "02713E6", "02713EZ", "02713F6", "02713FZ", "02713G6", "02713GZ", "02713T6", "02713TZ", "02713Z6", "02713ZZ", "0271446", "027144Z", "0271456", "027145Z", "0271466", "027146Z", "0271476", "027147Z", "02714D6", "02714DZ", "02714E6", "02714EZ", "02714F6", "02714FZ", "02714G6", "02714GZ", "02714T6", "02714TZ", "02714Z6", "02714ZZ", "0272046", "027204Z", "0272056", "027205Z", "0272066", "027206Z", "0272076", "027207Z", "02720D6", "02720DZ", "02720E6", "02720EZ", "02720F6", "02720FZ", "02720G6", "02720GZ", "02720T6", "02720TZ", "02720Z6", "02720ZZ", "0272346", "027234Z", "0272356", "027235Z", "0272366", "027236Z", "0272376", "027237Z", "02723D6", "02723DZ", "02723E6", "02723EZ", "02723F6", "02723FZ", "02723G6", "02723GZ", "02723T6", "02723TZ", "02723Z6", "02723ZZ", "0272446", "027244Z", "0272456", "027245Z", "0272466", "027246Z", "0272476", "027247Z", "02724D6", "02724DZ", "02724E6", "02724EZ", "02724F6", "02724FZ", "02724G6", "02724GZ", "02724T6", "02724TZ", "02724Z6", "02724ZZ", "0273046", "027304Z", "0273056", "027305Z", "0273066", "027306Z", "0273076", "027307Z", "02730D6", "02730DZ", "02730E6", "02730EZ", "02730F6", "02730FZ", "02730G6", "02730GZ", "02730T6", "02730TZ", "02730Z6", "02730ZZ", "0273346", "027334Z", "0273356", "027335Z", "0273366", "027336Z", "0273376", "027337Z", "02733D6", "02733DZ", "02733E6", "02733EZ", "02733F6", "02733FZ", "02733G6", "02733GZ", "02733T6", "02733TZ", "02733Z6", "02733ZZ", "0273446", "027344Z", "0273456", "027345Z", "0273466", "027346Z", "0273476", "027347Z", "02734D6", "02734DZ", "02734E6", "02734EZ", "02734F6", "02734FZ", "02734G6", "02734GZ", "02734T6", "02734TZ", "02734Z6", "02734ZZ" |
| Prior PCI | ICD-9-CM | "00.66", "36.04", "36.06", "36.07", "36.09", "V45.82" |
|  | SNOMED CT | "1086811000000105", "11101003", "149177005", "149255001", "175029007", "203741000000101", "211371000000105", "366879003", "36969009", "384693001", "609153008", "68466008", "752481000000106", "763725002" |
|  | ICD-10-CM | "Z95.5", "Z98.61", "T82.855" |
|  | SNOMED CT | "130541000119100", "316096004", "11018701000119109", "309786009", "316108005", "371808007", "371812001", "371822007", "371823002", "373108000", "405741001", "411481000000101", "418511000000100", "428308007", "428375006", "428664003", "428912006", "429245005", "473155009", "512081000000108", "371809004", "371811008", "251030009", "429245005" |
| Other coronary artery disease | ICD-10-CM | "I20", "I20.0", "I20.8", "I20.9", "I24.8", "I24.9", "I25.10", "I25.110", "I25.111", "I25.118", "I25.119", "I25.5", "I25.6", "I25.750", "I25.751", "I25.758", "I25.759", "I25.760", "I25.761", "I25.768", "I25.769", "I25.811", "I25.812", "I25.82", "I25.83", "I25.84", "I25.89", "I25.9", "I20", "I24", "I25", "I25.11", "I25.75", "I25.8", "I25.81" |
|  | SNOMED CT | "393587009", "394659003", "10365005", "117051000119103", "123641001", "123642008", "139011000119104", "155303000", "155305007", "155307004", "155309001", "155311005", "155312003", "155313008", "155314002", "155315001", "155316000", "155318004", "155322009", "15960141000119102", "1641000119107", "16754391000119100", "16891151000119103", "17828002", "19057007", "194795001", "194812005", "194815007", "194817004", "194818009", "194819001", "194821006", "194822004", "194826001", "194828000", "194829008", "194830003", "194831004", "194832006", "194833001", "194834007", "194835008", "194837000", "194838005", "194839002", "194840000", "194841001", "194842008", "194843003", "194849004", "194850004", "194851000", "194852007", "194853002", "194854008", "194855009", "194877008", "194878003", "195121002", "195540001", "195541002", "195543004", "195544005", "19830001000004106", "204379001", "21470009", "225566008", "233817007", "233818002", "233819005", "233821000", "233822007", "233823002", "233844002", "233970002", "23687008", "25106000", "2610009", "266231003", "266289009", "266290000", "266291001", "26900001", "271430002", "281091000", "28248000", "285141000119106", "285151000119108", "300995000", "315025001", "315348000", "32598000", "35928006", "361133006", "367416001", "371803003", "371804009", "371805005", "371806006", "371807002", "373144005", "373145006", "373146007", "39468009", "398274000", "408546009", "41334000", "413439005", "413838009", "413844008", "414024009", "414545008", "414795007", "41702007", "421327009", "429559004", "429673002", "442224005", "442240008", "442421004", "443502000", "444855007", "451041000124103", "451361000124102", "4557003", "455941000124104", "53741008", "59021001", "61490001", "63739005", "64333001", "6661000119101", "67682002", "719678003", "723862008", "724431008", "74218008", "75398000", "782699008", "78741000119103", "791000119109", "792842004", "827164008", "84537008", "85284003", "89323001", "8957000", "92517006", "10971000087107", "155320001", "176331000000107", "194824003", "194825002", "315026000", "414541000000105", "426856002", "46109009", "465021000000107", "471531000000105", "472100003", "603911000000102", "621571000000106", "621581000000108", "621601000000104", "621611000000102", "643861000000100", "643871000000107", "643891000000106", "646451000000105", "653031000000100", "653041000000109", "662031000000103", "671571000000105", "671581000000107", "712866001", "713405002", "82522008", "841141000000104", "854491000006109", "960181000006107" |
|  | ICD-9-CM | "411.89", "413.9", "414.00", "414.01", "414.06", "414.07", "414.2", "414.3", "414.4", "414.8", "414.9", "411", "411.8", "413", "414" |
| Peripheral arterial disease | ICD-10-CM | "I70.2", "I70.20", "I70.201", "I70.202", "I70.203", "I70.208", "I70.209", "I70.21", "I70.211", "I70.212", "I70.213", "I70.218", "I70.219", "I70.22", "I70.221", "I70.222", "I70.223", "I70.228", "I70.229", "I70.23", "I70.231", "I70.232", "I70.233", "I70.234", "I70.235", "I70.238", "I70.239", "I70.24", "I70.241", "I70.242", "I70.243", "I70.244", "I70.245", "I70.248", "I70.249", "I70.25", "I70.26", "I70.261", "I70.262", "I70.263", "I70.268", "I70.269", "I70.29", "I70.291", "I70.292", "I70.293", "I70.298", "I70.299", "I70.3", "I70.30", "I70.301", "I70.302", "I70.303", "I70.308", "I70.309", "I70.31", "I70.311", "I70.312", "I70.313", "I70.318", "I70.319", "I70.32", "I70.321", "I70.322", "I70.323", "I70.328", "I70.329", "I70.33", "I70.331", "I70.332", "I70.333", "I70.334", "I70.335", "I70.338", "I70.339", "I70.34", "I70.341", "I70.342", "I70.343", "I70.344", "I70.345", "I70.348", "I70.349", "I70.35", "I70.36", "I70.361", "I70.362", "I70.363", "I70.368", "I70.369", "I70.39", "I70.391", "I70.392", "I70.393", "I70.398", "I70.399", "I70.4", "I70.40", "I70.401", "I70.402", "I70.403", "I70.408", "I70.409", "I70.41", "I70.411", "I70.412", "I70.413", "I70.418", "I70.419", "I70.42", "I70.421", "I70.422", "I70.423", "I70.428", "I70.429", "I70.43", "I70.431", "I70.432", "I70.433", "I70.434", "I70.435", "I70.438", "I70.439", "I70.44", "I70.441", "I70.442", "I70.443", "I70.444", "I70.445", "I70.448", "I70.449", "I70.45", "I70.46", "I70.461", "I70.462", "I70.463", "I70.468", "I70.469", "I70.49", "I70.491", "I70.492", "I70.493", "I70.498", "I70.499", "I70.5", "I70.50", "I70.501", "I70.502", "I70.503", "I70.508", "I70.509", "I70.51", "I70.511", "I70.512", "I70.513", "I70.518", "I70.519", "I70.52", "I70.521", "I70.522", "I70.523", "I70.528", "I70.529", "I70.53", "I70.531", "I70.532", "I70.533", "I70.534", "I70.535", "I70.538", "I70.539", "I70.54", "I70.541", "I70.542", "I70.543", "I70.544", "I70.545", "I70.548", "I70.549", "I70.55", "I70.56", "I70.561", "I70.562", "I70.563", "I70.568", "I70.569", "I70.59", "I70.591", "I70.592", "I70.593", "I70.598", "I70.599", "I70.6", "I70.60", "I70.601", "I70.602", "I70.603", "I70.608", "I70.609", "I70.61", "I70.611", "I70.612", "I70.613", "I70.618", "I70.619", "I70.62", "I70.621", "I70.622", "I70.623", "I70.628", "I70.629", "I70.63", "I70.631", "I70.632", "I70.633", "I70.634", "I70.635", "I70.638", "I70.639", "I70.64", "I70.641", "I70.642", "I70.643", "I70.644", "I70.645", "I70.648", "I70.649", "I70.65", "I70.66", "I70.661", "I70.662", "I70.663", "I70.668", "I70.669", "I70.69", "I70.691", "I70.692", "I70.693", "I70.698", "I70.699", "I70.7", "I70.70", "I70.701", "I70.702", "I70.703", "I70.708", "I70.709", "I70.71", "I70.711", "I70.712", "I70.713", "I70.718", "I70.719", "I70.72", "I70.721", "I70.722", "I70.723", "I70.728", "I70.729", "I70.73", "I70.731", "I70.732", "I70.733", "I70.734", "I70.735", "I70.738", "I70.739", "I70.74", "I70.741", "I70.742", "I70.743", "I70.744", "I70.745", "I70.748", "I70.749", "I70.75", "I70.76", "I70.761", "I70.762", "I70.763", "I70.768", "I70.769", "I70.79", "I70.791", "I70.792", "I70.793", "I70.798", "I70.799", "I70.92", "I75.0", "I73.9", “I.75", "I75.0", "I75.01", "I75.011", "I75.012", "I75.013", "I75.019", "I75.02", "I75.021", "I75.022", "I75.023", "I75.029", "I75.8", "I75.81", "I75.89" |
|  | ICD-9-CM | "440.4", "440.21", "440.2", "440.21", "440.22", "440.23", "440.24", "440.29", "440.3", "440.31", "440.32", "440.4", "445", "445", "445.0", "445.01", "445.02", "445.8", "445.81", "445.89" |
|  | SNOMED CT | "10666031000119100", "12236951000119108", "12237071000119100", "12237191000119103", "13954005", "155430009", "155431008", "15614009", "195312007", "201250006", "201253008", "233956002", "238793001", "238794007", "238795008", "266320008", "284911000119108", "429768000", "5561000119107", "63491006", "8001000119106", "275520000", "15966701000119103", "15966741000119101", "15966781000119106", "16009351000119100", "16009391000119105", "16012471000119105", "16012631000119105", "16012711000119107", "51677000" |
| Prior peripheral revascularization | ICD-10-CM | "I70.3", "I70.30", "I70.301", "I70.302", "I70.303", "I70.308", "I70.309", "I70.31", "I70.311", "I70.312", "I70.313", "I70.318", "I70.319", "I70.32", "I70.321", "I70.322", "I70.323", "I70.328", "I70.329", "I70.33", "I70.331", "I70.332", "I70.333", "I70.334", "I70.335", "I70.338", "I70.339", "I70.34", "I70.341", "I70.342", "I70.343", "I70.344", "I70.345", "I70.348", "I70.349", "I70.35", "I70.36", "I70.361", "I70.362", "I70.363", "I70.368", "I70.369", "I70.39", "I70.391", "I70.392", "I70.393", "I70.398", "I70.399", "I70.4", "I70.40", "I70.401", "I70.402", "I70.403", "I70.408", "I70.409", "I70.41", "I70.411", "I70.412", "I70.413", "I70.418", "I70.419", "I70.42", "I70.421", "I70.422", "I70.423", "I70.428", "I70.429", "I70.43", "I70.431", "I70.432", "I70.433", "I70.434", "I70.435", "I70.438", "I70.439", "I70.44", "I70.441", "I70.442", "I70.443", "I70.444", "I70.445", "I70.448", "I70.449", "I70.45", "I70.46", "I70.461", "I70.462", "I70.463", "I70.468", "I70.469", "I70.49", "I70.491", "I70.492", "I70.493", "I70.498", "I70.499", "I70.5", "I70.50", "I70.501", "I70.502", "I70.503", "I70.508", "I70.509", "I70.51", "I70.511", "I70.512", "I70.513", "I70.518", "I70.519", "I70.52", "I70.521", "I70.522", "I70.523", "I70.528", "I70.529", "I70.53", "I70.531", "I70.532", "I70.533", "I70.534", "I70.535", "I70.538", "I70.539", "I70.54", "I70.541", "I70.542", "I70.543", "I70.544", "I70.545", "I70.548", "I70.549", "I70.55", "I70.56", "I70.561", "I70.562", "I70.563", "I70.568", "I70.569", "I70.59", "I70.591", "I70.592", "I70.593", "I70.598", "I70.599", "Z95.820", "Z95.828", "Z95.9", "I70.3", "I70.30", "I70.301", "I70.302", "I70.303", "I70.308", "I70.309", "I70.31", "I70.311", "I70.312", "I70.313", "I70.318", "I70.319", "I70.32", "I70.321", "I70.322", "I70.323", "I70.328", "I70.329", "I70.33", "I70.331", "I70.332", "I70.333", "I70.334", "I70.335", "I70.338", "I70.339", "I70.34", "I70.341", "I70.342", "I70.343", "I70.344", "I70.345", "I70.348", "I70.349", "I70.35", "I70.36", "I70.361", "I70.362", "I70.363", "I70.368", "I70.369", "I70.39", "I70.391", "I70.392", "I70.393", "I70.398", "I70.399", "I70.4", "I70.40", "I70.401", "I70.402", "I70.403", "I70.408", "I70.409", "I70.41", "I70.411", "I70.412", "I70.413", "I70.418", "I70.419", "I70.42", "I70.421", "I70.422", "I70.423", "I70.428", "I70.429", "I70.43", "I70.431", "I70.432", "I70.433", "I70.434", "I70.435", "I70.438", "I70.439", "I70.44", "I70.441", "I70.442", "I70.443", "I70.444", "I70.445", "I70.448", "I70.449", "I70.45", "I70.46", "I70.461", "I70.462", "I70.463", "I70.468", "I70.469", "I70.49", "I70.491", "I70.492", "I70.493", "I70.498", "I70.499", "I70.5", "I70.50", "I70.501", "I70.502", "I70.503", "I70.508", "I70.509", "I70.51", "I70.511", "I70.512", "I70.513", "I70.518", "I70.519", "I70.52", "I70.521", "I70.522", "I70.523", "I70.528", "I70.529", "I70.53", "I70.531", "I70.532", "I70.533", "I70.534", "I70.535", "I70.538", "I70.539", "I70.54", "I70.541", "I70.542", "I70.543", "I70.544", "I70.545", "I70.548", "I70.549", "I70.55", "I70.56", "I70.561", "I70.562", "I70.563", "I70.568", "I70.569", "I70.59", "I70.591", "I70.592", "I70.593", "I70.598", "I70.599", "I70.6", "I70.60", "I70.601", "I70.602", "I70.603", "I70.608", "I70.609", "I70.61", "I70.611", "I70.612", "I70.613", "I70.618", "I70.619", "I70.62", "I70.621", "I70.622", "I70.623", "I70.628", "I70.629", "I70.63", "I70.631", "I70.632", "I70.633", "I70.634", "I70.635", "I70.638", "I70.639", "I70.64", "I70.641", "I70.642", "I70.643", "I70.644", "I70.645", "I70.648", "I70.649", "I70.65", "I70.66", "I70.661", "I70.662", "I70.663", "I70.668", "I70.669", "I70.69", "I70.691", "I70.692", "I70.693", "I70.698", "I70.699", "I70.7", "I70.70", "I70.701", "I70.702", "I70.703", "I70.708", "I70.709", "I70.71", "I70.711", "I70.712", "I70.713", "I70.718", "I70.719", "I70.72", "I70.721", "I70.722", "I70.723", "I70.728", "I70.729", "I70.73", "I70.731", "I70.732", "I70.733", "I70.734", "I70.735", "I70.738", "I70.739", "I70.74", "I70.741", "I70.742", "I70.743", "I70.744", "I70.745", "I70.748", "I70.749", "I70.75", "I70.76", "I70.761", "I70.762", "I70.763", "I70.768", "I70.769", "I70.79", "I70.791", "I70.792", "I70.793", "I70.798", "I70.799" |
|  | ICD-9-CM | "V45.89", "440.3", "440.3", "440.31", "440.32" |
|  | SNOMED CT | "10988321000119100", "130041000119106", "308071004", "8001000119106" |
| Abdominal aortic aneurysm | ICD-10-CM | "I70.0" |
|  | ICD-9-CM | "440.0" |
|  | SNOMED CT | "155415000", "195252007", "233955003", "233956002", "81817003" |
| Prior stroke | ICD-9-CM | "438", "438.0", "438", "438.0", "438.10", "438.1", "438.10", "438.11", "438.11", "438.12", "438.12", "438.13", "438.13", "438.14", "438.14", "438.19", "438.19", "438.20", "438.2", "438.20", "438.21", "438.21", "438.22", "438.22", "438.30", "438.3", "438.30", "438.31", "438.31", "438.32", "438.32", "438.40", "438.4", "438.40", "438.41", "438.41", "438.42", "438.42", "438.50", "438.5", "438.50", "438.51", "438.51", "438.52", "438.52", "438.53", "438.53", "438.6", "438.6", "438.7", "438.7", "438.8", "438.81", "438.81", "438.82", "438.82", "438.83", "438.83", "438.84", "438.84", "438.85", "438.85", "438.89", "438.89", "438.9", "438.9", "V12.54" |
|  | SNOMED CT | "138778001", "138787005", "161506002", "161518006", "195243003", "195245005", "271395009", "275526006", "275527002", "308067002", "315757009", "425642008", "425882004", "426033005", "426788002", "427065003", "427926004", "428668000", "429235008", "429993008", "430947007", "430959006", "433183000", "440140008", "441894009", "441960006", "441991000", "442181008", "442212003", "442617003", "442733008", "699429007", "723083001", "361000119103", "1131000119105", "23671000119107", "33301000119105", "33331000119103", "40161000119102", "46421000119102", "90921000119104", "91601000119109", "92341000119107", "97531000119106", "99051000119101", "102831000119104", "103761000119107", "106241000119108", "108691000119102", "118951000119103", "118961000119101", "118971000119107", "134771000119108", "137991000119103", "140281000119108", "141281000119101", "141821000119104", "141831000119101", "145741000119101", "148871000119109", "290891000119100", "290901000119101", "290911000119103", "290921000119105", "291081000119100", "291101000119107", "413751000000100", "417791000000102", "428011000000100", "432051000124108", "672371000119103", "672381000119100", "672421000119109", "672481000119108", "674131000119105", "690051000119100", "690181000119108", "690191000119106", "690231000119102", "690241000119106", "690251000119108", "690261000119105", "690281000119101" |
|  | ICD-10-CM | "I69.3", "I69.3", "I69.30", "I69.30", "I69.31", "I69.31", "I69.310", "I69.310", "I69.311", "I69.311", "I69.312", "I69.312", "I69.313", "I69.313", "I69.314", "I69.314", "I69.315", "I69.315", "I69.318", "I69.318", "I69.319", "I69.319", "I69.32", "I69.32", "I69.320", "I69.320", "I69.321", "I69.321", "I69.322", "I69.322", "I69.323", "I69.323", "I69.328", "I69.328", "I69.33", "I69.33", "I69.331", "I69.331", "I69.332", "I69.332", "I69.333", "I69.333", "I69.334", "I69.334", "I69.339", "I69.339", "I69.34", "I69.34", "I69.341", "I69.341", "I69.342", "I69.342", "I69.343", "I69.343", "I69.344", "I69.344", "I69.349", "I69.349", "I69.35", "I69.35", "I69.351", "I69.351", "I69.352", "I69.352", "I69.353", "I69.353", "I69.354", "I69.354", "I69.359", "I69.359", "I69.36", "I69.36", "I69.361", "I69.361", "I69.362", "I69.362", "I69.363", "I69.363", "I69.364", "I69.364", "I69.365", "I69.365", "I69.369", "I69.369", "I69.39", "I69.39", "I69.390", "I69.390", "I69.391", "I69.391", "I69.392", "I69.392", "I69.393", "I69.393", "I69.398", "I69.398", "Z86.73" |
| Acute stroke | ICD-9-CM | "433.01", "433.11", "433.21", "433.31", "433.81", "433.91", "434.01", "434.11", "434.91" |
|  | SNOMED CT | "81037000", "195185009", "195186005", "195189003", "195190007", "195230003", "195247002", "195599001", "195600003", "230698000", "705128004", "705130002", "419691000000103", "419701000000103", "433931000124109", "444151000000109" |
|  | ICD-10-CM | "I63", "I63.0", "I63.00", "I63.01", "I63.011", "I63.012", "I63.013", "I63.019", "I63.02", "I63.03", "I63.031", "I63.032", "I63.033", "I63.039", "I63.09", "I63.1", "I63.10", "I63.11", "I63.111", "I63.112", "I63.113", "I63.119", "I63.12", "I63.13", "I63.131", "I63.132", "I63.133", "I63.139", "I63.19", "I63.2", "I63.20", "I63.21", "I63.211", "I63.212", "I63.213", "I63.219", "I63.22", "I63.23", "I63.231", "I63.232", "I63.233", "I63.239", "I63.29", "I63.3", "I63.30", "I63.31", "I63.311", "I63.312", "I63.313", "I63.319", "I63.32", "I63.321", "I63.322", "I63.323", "I63.329", "I63.33", "I63.331", "I63.332", "I63.333", "I63.339", "I63.34", "I63.341", "I63.342", "I63.343", "I63.349", "I63.39", "I63.4", "I63.40", "I63.41", "I63.411", "I63.412", "I63.413", "I63.419", "I63.42", "I63.421", "I63.422", "I63.423", "I63.429", "I63.43", "I63.431", "I63.432", "I63.433", "I63.439", "I63.44", "I63.441", "I63.442", "I63.443", "I63.449", "I63.49", "I63.5", "I63.50", "I63.51", "I63.511", "I63.512", "I63.513", "I63.519", "I63.52", "I63.521", "I63.522", "I63.523", "I63.529", "I63.53", "I63.531", "I63.532", "I63.533", "I63.539", "I63.54", "I63.541", "I63.542", "I63.543", "I63.549", "I63.59", "I63.6", "I63.8", "I63.81", "I63.89", "I63.9" |
| Prior TIA | ICD-10-CM | "Z86.73" |
|  | ICD-9-CM | "V12.54" |
|  | SNOMED CT | "13016361000119101", "138782004", "140221000119109", "161511000", "836061000000108", "836071000000101" |
| Acute TIA | ICD-9-CM | "G45", "435", "435.0", "435.1", "435.3", "435.8", "435.9" |
|  | SNOMED CT | "29322000", "34781003", "64009001", "155404005", "194495002", "195196001", "195197005", "195198000", "195199008", "195201005", "195204002", "195209007", "195210002", "195211003", "195222004", "266314007", "394517009", "467911000000109", "639511000000102" |
|  | ICD-10-CM | "G45.0", "G45.2", "G45.8", "G45.9", "G46.0", "G46.1", "G46.2", "I67.81" |
| Other cerebrovascular disease | ICD-9-CM | "433.00", "433.20", "433.30", "433.80", "433.90" |
|  | SNOMED CT | "1055001", "28790007", "43658003", "54519002", "64586002", "67992007", "69798007", "73192008", "78658006", "90520006", "155395002", "155396001", "155397005", "155398000", "155399008", "195180004", "195181000", "195182007", "195183002", "195184008", "195187001", "195200006", "233963002", "233964008", "266253001", "266254007", "371158002", "426651005", "703180005", "703184001", "703206009", "787044009", "21000119103", "9611000119107", "285161000119105", "285171000119104", "285191000119103", "285201000119100", "430721000124101", "430731000124103", "430781000124102", "430831000124106", "430841000124101", "430861000124102", "433821000124102", "433921000124106", "435271000124103", "435281000124100", "436021000124100", "584151000000106", "584161000000109", "27820001000004104" |
|  | ICD-10-CM | "G45.1", "I65", "I65.0", "I65.01", "I65.02", "I65.03", "I65.09", "I65.1", "I65.2", "I65.21", "I65.22", "I65.23", "I65.29", "I65.8", "I65.9", "I66", "I66.0", "I66.01", "I66.02", "I66.03", "I66.09", "I66.1", "I66.11", "I66.12", "I66.13", "I66.19", "I66.2", "I66.21", "I66.22", "I66.23", "I66.29", "I66.3", "I66.8", "I66.9", "I67.2", "I67.82" |
| **Diabetes** | ICD-10-CM | 'E11.3312', 'E11.3529', 'E11.3522', 'E11.22', 'E11.3523', 'E11.3392', 'E11.3491', 'E11.3492', 'E11.41', 'E11.3533', 'E11.21', 'E11.3513', 'E11.3559', 'E11.42', 'E11.622', 'E11.01', 'E11.3511', 'E11.3399', 'E11.3313', 'E11.3493', 'E11.3543', 'E11.3592', 'E11.3521', 'E11.49', 'E11.319', 'E11.3412', 'E11.3293', 'E11.3411', 'E11.630', 'E11.3532', 'E11.3419', 'E11.3291', 'E11.3292', 'E11.3512', 'E11.3519', 'E11.618', 'E11.52', 'E11.638', 'E11.641', 'O24.119', 'E11.8', 'E11.3212', 'E11.628', 'E11.3213', 'E11.65', 'E11.11', 'E11.3311', 'E11.3393', 'E11.37X9', 'E11.610', 'E11.9', 'E11.621', 'E11.29', 'E11.3542', 'E11.3541', 'O24.13', 'E11.311', 'E11.649', 'E11.3551', 'E11.69', 'E11.10', 'E11.3299', 'E11.3499', 'E11.36', 'E11.37X3', 'E11.43', 'E11.59', 'E11.3319', 'E11.3552', 'O24.113', 'E11.3391', 'E11.3211', 'E11.37X2', 'E11.37X1', 'E11.3219', 'O24.112', 'E11.3539', 'E11.3599', 'E11.40', 'E11.3593', 'O24.111', 'E11.3413', 'E11.3553', 'E11.3591', 'E11.44', 'E11.00', 'O24.12', 'E11.3549', 'E11.620', 'E11.51', 'E11.39', 'E11.3531' |
|  | ICD-9-CM | '250.62', '250.9', '250.82', '250.8', '250.2', '250.72', '250.1', '250.42', '250.5', '250.32', '250.12', '250.3', '250.92', '250.7', '250', '250.52', '250.6', '250.02', '250.22', '250.4' |
|  | SNOMED CT | '120731000119103', '421326000', '10656271000119100', '111231000119109', '157141000119108', '711000119100', '137931000119102', '237599002', '44054006', '87921000119104', '781000119106', '421750000', '12811000119100', '427027005', '701000119103', '751000119104', '1521000119100', '127991000119101', '1531000119102', '713706002', '190351004', '71421000119105', '314902007', '443694000', '10661671000119100', '90791000119104', '713703005', '761000119102', '72061000119104', '190388001', '190392008', '424989000', '422099009', '712883005', '1481000119100', '201000119106', '741000119101', '368051000119109', '609567009', '190390000', '84361000119102', '791000119109', '422034002', '164971000119101', '138921000119104', '313436004', '1501000119109', '81531005', '1541000119106', '190331003', '368581000119106', '138941000119105', '119831000119106', '10660471000119100', '314903002', '422014003', '140391000119101', '1511000119107', '359638003', '422166005', '90781000119102', '359642000', '395204000', '190346005', '97341000119105', '721000119107', '87451000119102', '428007007', '420279001', '237627000', '771000119108', '138911000119106', '444110003', '421707005', '314904008', '190389009', '423263001', '731000119105', '190356009' |
| **Retinopathy** | ICD-9 | 250.5', '250.50', '250.51', '250.52', '250.53', '362.00', '362.01', '362.02', '362.03', '362.04', '362.05', '362.06', '362.07' |
|  | ICD-10-CM | ‘E10.3', 'E10.31', 'E10.311', 'E10.319', 'E10.32', 'E10.321', 'E10.3211', 'E10.3212', 'E10.3213', 'E10.3219', 'E10.329', 'E10.3291', 'E10.3292', 'E10.3293', 'E10.3299', 'E10.33', 'E10.331', 'E10.3311', 'E10.3312', 'E10.3313', 'E10.3319', 'E10.339', 'E10.3391', 'E10.3392', 'E10.3393', 'E10.3399', 'E10.34', 'E10.341', 'E10.3411', 'E10.3412', 'E10.3413', 'E10.3419', 'E10.349', 'E10.3491', 'E10.3492', 'E10.3493', 'E10.3499', 'E10.35', 'E10.351', 'E10.3511', 'E10.3512', 'E10.3513', 'E10.3519', 'E10.352', 'E10.3521', 'E10.3522', 'E10.3523', 'E10.3529', 'E10.353', 'E10.3531', 'E10.3532', 'E10.3533', 'E10.3539', 'E10.354', 'E10.3541', 'E10.3542', 'E10.3543', 'E10.3549', 'E10.355', 'E10.3551', 'E10.3552', 'E10.3553', 'E10.3559', 'E10.359', 'E10.3591', 'E10.3592', 'E10.3593', 'E10.3599', 'E10.36', 'E10.37', 'E10.37X1', 'E10.37X2', 'E10.37X3', 'E10.37X9', 'E10.39', 'E11.3', 'E11.31', 'E11.311', 'E11.319', 'E11.32', 'E11.321', 'E11.3211', 'E11.3212', 'E11.3213', 'E11.3219', 'E11.329', 'E11.3291', 'E11.3292', 'E11.3293', 'E11.3299', 'E11.33', 'E11.331', 'E11.3311', 'E11.3312', 'E11.3313', 'E11.3319', 'E11.339', 'E11.3391', 'E11.3392', 'E11.3393', 'E11.3399', 'E11.34', 'E11.341', 'E11.3411', 'E11.3412', 'E11.3413', 'E11.3419', 'E11.349', 'E11.3491', 'E11.3492', 'E11.3493', 'E11.3499', 'E11.35', 'E11.351', 'E11.3511', 'E11.3512', 'E11.3513', 'E11.3519', 'E11.352', 'E11.3521', 'E11.3522', 'E11.3523', 'E11.3529', 'E11.353', 'E11.3531', 'E11.3532', 'E11.3533', 'E11.3539', 'E11.354', 'E11.3541', 'E11.3542', 'E11.3543', 'E11.3549', 'E11.355', 'E11.3551', 'E11.3552', 'E11.3553', 'E11.3559', 'E11.359', 'E11.3591', 'E11.3592', 'E11.3593', 'E11.3599', 'E11.36', 'E11.37', 'E11.37X1', 'E11.37X2', 'E11.37X3', 'E11.37X9', 'E11.39' |
| **Rheumatoid arthritis** | ICD-9 | '714.0', '714.1', '714.2', '714.30', '714.31', '714.32', '714.33', '714.4', '714.81' |
|  | ICD-10-CM | 'M06.9', 'M05.00', 'M05.30', 'M05.60', 'M06.1', 'M08.00', 'M08.3', 'M08.40', 'M12.00', 'M05.10' |
| **Psoriasis** | ICD-9 | '696.0', '696.1' |
|  | ICD-10-CM | 'L40.59', 'L40.9', 'L40.0', 'L40.1', 'L40.2', 'L40.3', 'L40.8' |
| **HIV** | ICD-9 | '042' |
|  | ICD-10-CM | 'B20' |
| **Neuropathy** | ICD-9 | '249.6', '250.6', '250.60', '250.61', '250.62', '250.63', '357.2', '337.1' |
|  | ICD-10-CM | 'E10.4', 'E10.40', 'E10.41', 'E10.42', 'E10.43', 'E10.44', 'E10.49', 'E11.4', 'E11.40', 'E11.41', 'E11.42', 'E11.43', 'E11.44', 'E11.49' |
| **Hepatitis C** | ICD-9 | '070.51', '070.41', 'V02.62', '070.54', '070.70', '070.71' |
|  | ICD-10-CM | 'B17.10', 'B17.11', 'Z22.52', 'B18.12', 'B19.20', 'B19.21' |

ASCVD, atherosclerotic cardiovascular disease; CABG, coronary artery bypass graft; CM, clinical modification; CPT, Current Procedural Terminology; HCPCS, Healthcare Common Procedures Coding System; HIV, human immunodeficiency virus; ICD-9, International Classification of Diseases, 9th Revision; ICD-10, International Classification of Diseases, 10th Revision; PCI, percutaneous coronary intervention; PCS, Procedure Coding System; SNOMED CT, Systematized Nomenclature of Medicine Clinical Terms; TIA, transient ischemic attack.

**Table S2. Factors Associated with High-Intensity Statin Use in Univariable Modeling**

| **Characteristic** | **Odds ratio (95% CI)** |
| --- | --- |
| **Age, years (Ref: 40–54)** |  |
| 55–64 | 1.50 (1.46–1.54) |
| 65 to <75 | 1.73 (1.69–1.78) |
| **Sex (Ref: male)** |  |
| Female | 0.86 (0.84–0.88) |
| **Race (Ref: White)** |  |
| American Indian or Alaska Native | 0.71 (0.63–0.81) |
| Black or African American | 1.07 (1.04–1.10) |
| Mixed | 0.85 (0.70–1.02) |
| Other | 0.97 (0.91–1.03) |
| **Ethnicity (Ref: not Hispanic or Latino)** |  |
| Hispanic or Latino | 0.86 (0.83–0.89) |
| **Inflammatory Conditions** |  |
| Rheumatoid arthritis | 0.79 (0.73–0.86) |
| Psoriasis | 0.85 (0.78–0.93) |
| HIV | 0.84 (0.72–0.98) |
| Hepatitis C | 0.45 (0.41–0.49) |
| **Hypertension** | 1.67 (1.63–1.71) |
| **Diabetes‑specific risk enhancers** |  |
| Retinopathy | 1.32 (1.24–1.39) |
| Neuropathy | 1.10 (1.07–1.14) |
| **CKD (Ref: normal eGFR >90 mL/min/1.73m^2^)** |  |
| Stage 2 (eGFR, 60–89 mL/min/1.73m^2^) | 1.21 (1.19–1.24) |
| Stage 3 (eGFR, 30–59 mL/min/1.73m^2^) | 1.42 (1.38–1.48) |
| Stage 4–5 (eGFR, <30 mL/min/1.73m^2^) | 1.31 (1.22–1.42) |

ASCVD, atherosclerotic cardiovascular disease; CKD, chronic kidney disease; eGFR, estimated glomerular filtration rate; HIV, human immunodeficiency virus; Ref, reference. For comorbidities, reference category is absence of condition.

**Table S3. Changes in LDL-C Among Patients with LDL-C Measurements at Baseline and 1-Year Follow-up**

| **Characteristic** | **Overall**  *N* = 89,120 | **LDL-C <70 mg/dL at baseline**  *n* = 25,032 | **LDL-C ≥70 mg/dL at baseline**  *n* = 64,088 |
| --- | --- | --- | --- |
| **LDL-C at baseline, median (IQR), mg/dL** | 87.0 (67.0–112.0) | 56.0 (46.0–63.0) | 100.0 (84.0–123.0) |
| **LDL-C at 1 year, median (IQR), mg/dL** | 82.0 (63.0–106.0) | 60.0 (48.0–73.0) | 92.0 (74.0–114.0) |
| **Absolute Change in LDL-C, median (IQR), mg/dL** | −3.0 (−18.0 to 11.0) | 5.0 (−5.0 to 17.0) | −7.0 (−25.0 to 7.0) |
| **Percent Change in LDL-C, median (IQR)** | 0.0 (−0.2 to 0.0) | 0.0 (−0.1 to 0.0) | 0.0 (−0.2 to 0.0) |
| **LDL-C at baseline, *n* (%)** |  |  |  |
| <70 mg/dL | 25,032 (28.1) | 25,032 (100.0) | 0 (0.0) |
| 70–99 mg/dL | 31,718 (35.6) | 0 (0.0) | 31,718 (49.5) |
| ≥100 mg/dL | 32,370 (36.3) | 0 (0.0) | 32,370 (50.5) |
| **LDL-C at 1 year, *n* (%)** |  |  |  |
| <70 mg/dL | 29,487 (33.1) | 17,435 (69.7) | 12,052 (18.8) |
| 70–99 mg/dL | 32,492 (36.5) | 6133 (24.5) | 26,359 (41.1) |
| ≥100 mg/dL | 27,141 (30.5) | 1464 (5.8) | 25,677 (40.1) |

IQR, interquartile range; LDL-C, low-density lipoprotein cholesterol.

**Table S4. Changes in LDL-C Among Patients with LDL-C Measurements at Baseline and 1-Year Follow-up by LDL-C Categories**

| **Characteristic** | **LDL-C at follow-up** | | | |
| --- | --- | --- | --- | --- |
|  | **Overall**,  *N* = 89,120 | **<70 mg/dL**,  *n* = 29,487 | **70–99 mg/dL**,  *n* = 32,492 | ≥**100 mg/dL**,  *n* = 27,141 |
| **LDL-C at baseline, *n* (%)** |  |  |  |  |
| <70 mg/dL | 25,032 (28.1) | 17,435 (59.1) | 6133 (18.9) | 1464 (5.4) |
| 70–99 mg/dL | 31,718 (35.6) | 8087 (27.4) | 17,102 (52.6) | 6529 (24.1) |
| ≥100 mg/dL | 32,370 (36.3) | 3965 (13.4) | 9257 (28.5) | 19,148 (70.6) |

LDL-C, low-density lipoprotein cholesterol.

**Figure S1. Age and Sex-Specific Predicted Probability of Moderate- to High‑Intensity Statin Utilization in Patients with Diabetes**

**
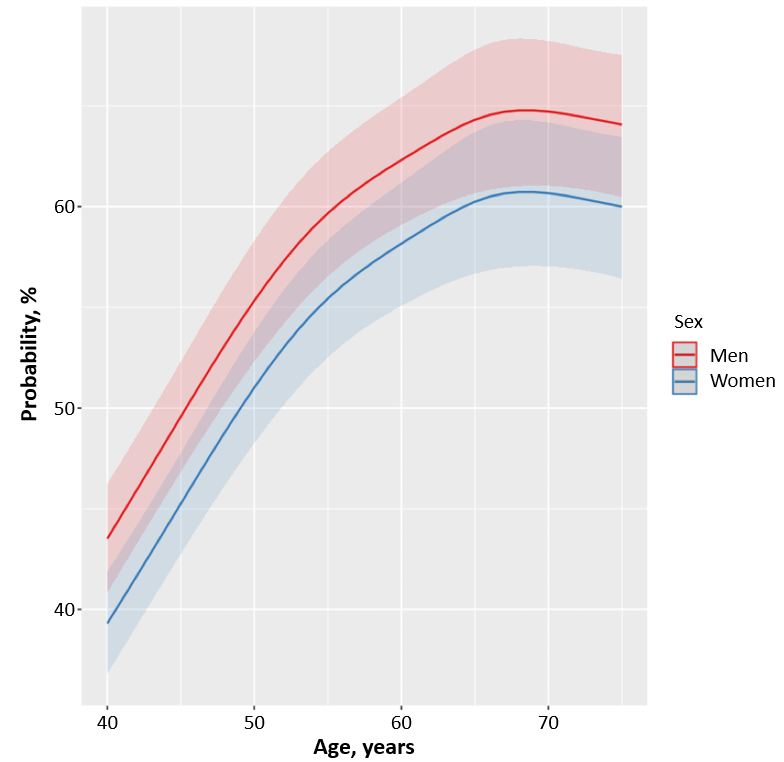
**

**Figure S2. Utilization of Non-Statin Lipid‑Lowering Therapy at Baseline.**

**PCSK9i, proprotein convertase subtilisin–kexin type 9.**

**
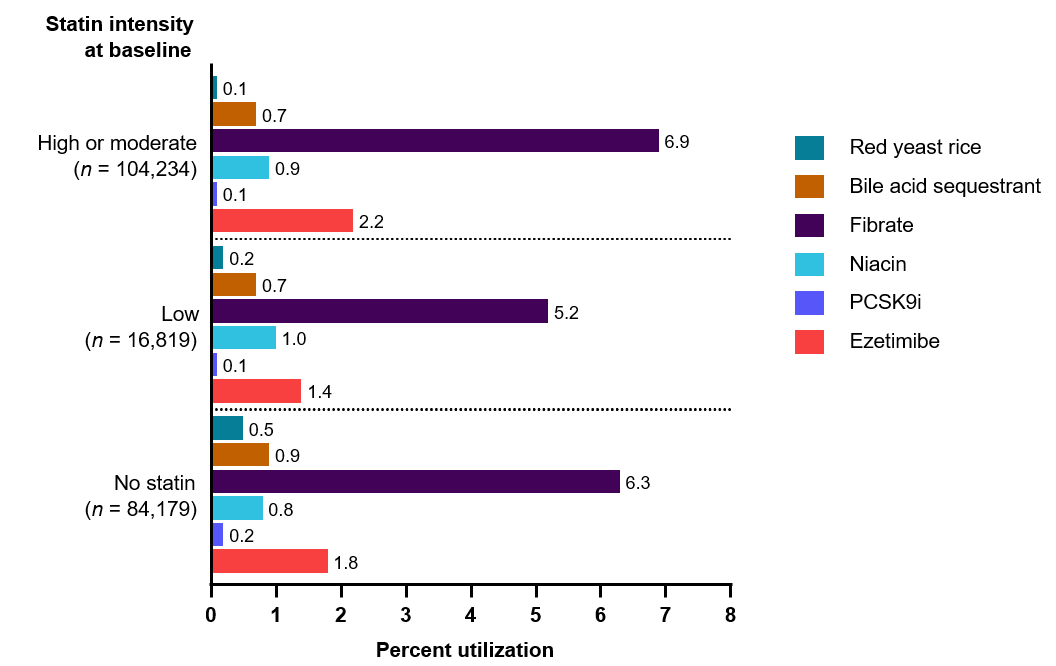
**
